# A Tutorial on Best Practices and Pitfalls in Applying Machine Learning to Environmental Research

**DOI:** 10.1021/acsenvironau.6c00025

**Published:** 2026-06-05

**Authors:** Zidong Yan, Jiaqi Li, Weican Zhang, Haonan Wen, Hao Yu, Miao Yu, Qian Liu, Guibin Jiang

**Affiliations:** † State Key Laboratory of Environmental Chemistry and Toxicology, 74519 Research Center for Eco-Environmental Sciences, Chinese Academy of Sciences, Beijing 100085, China; ‡ College of Resources and Environment, University of Chinese Academy of Sciences, Beijing 100049, China; § The Jackson Laboratory, 10 Discovery Drive, Farmington, Connecticut 06032, United States; ∥ Institute of Environment and Health, Jianghan University, Wuhan 430056, China

**Keywords:** Machine learning, Environmental research, Domain knowledge, Modeling workflow, Model evaluation, Methodological pitfalls, Model interpretability, Environmental decision-making, Causal machine learning

## Abstract

Machine learning (ML) has become a powerful paradigm for extracting structures from complex environmental data and supporting scientific inference across diverse subfields. Its potential, however, is often limited by gaps in the appropriate application of domain knowledge to machine-learning workflows, variability in data quality, and methodological choices that can distort model behavior or its interpretation. This Tutorial provides practical guidance on how domain expertise can be effectively integrated into the design of environmentally meaningful machine learning models and outlines a coherent workflow that integrates crucial stages, including data preprocessing, model development, evaluation, and interpretability. It also examines recurring pitfalls that arise along this pipeline and explains how they shape the credibility and reliability of machine-learning findings in environmental contexts. By consolidating these principles, this Tutorial aims to provide researchers with a clearer foundation for using machine learning in ways that are scientifically grounded, methodologically rigorous, and better aligned with the needs of environmental decision-making.

## Graphical abstract

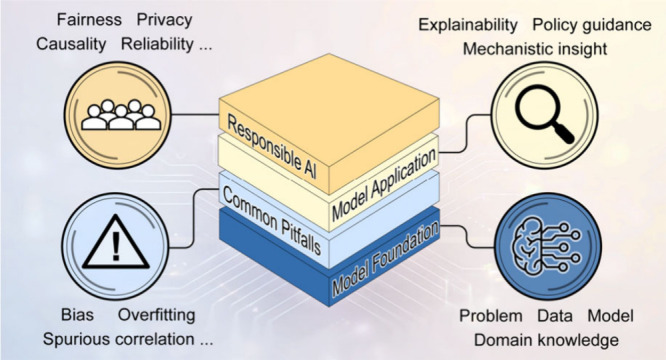

## Introduction

Environmental research is entering an “era of big data”, characterized by unprecedented data availability. Observations from laboratory experiments, *in situ* monitoring networks, and remote sensing platforms generate vast, heterogeneous datasets spanning spatial and temporal scales from molecular to global.^1^ Consequently, the primary challenge is increasingly shifting from data collection to knowledge synthesis. However, a substantial portion of environmental research still relies on relatively small and often costly datasets. In such settings, data-driven approaches can help extract signal from noisy observations, leverage spatiotemporal structure, and make more efficient use of limited data. Machine learning (ML) provides flexible tools for capturing complex, nonlinear relationships, including physical or empirical relationships that are difficult to explicitly parametrize in traditional models. This capability can improve predictive performance in environmental applications. When combined with appropriate interpretability methods, it may also offer insights into underlying processes. As a result, ML has driven major advances in both fundamental process understanding and applied predictive capability within environmental science.^2,3^

Despite these advances, the widespread adoption of MLs introduces new challenges. In environmental applications, where ML outputs are increasingly used to inform policy and risk management,^4–9^ the reliability of model-based decisions depends critically on data quality, representativeness, and appropriate model evaluation. The limited interpretability of many ML approaches can further hinder the assessment of whether learned relationships are physically meaningful. Addressing these challenges requires careful integration of data evaluation, domain knowledge, and transparent modeling practices to support robust environmental decision-making.

This Tutorial aims to bridge the methodological and conceptual gap between environmental science and machine learning. Unlike existing guideline-oriented reviews, this Tutorial emphasizes practical implementation through step-by-step workflows, reproducible code examples, and the integration of empirical validation. It begins by emphasizing the importance of domain-specific prior knowledge across different environmental disciplines, followed by an overview of the fundamental principles and workflows of machine learning. This Tutorial further addresses critical aspects of model interpretability and common pitfalls encountered across the modeling workflow, from initial data acquisition to result interpretation, and finally outlines key directions for future research. As a tutorial-style overview, this work offers essential guidance on how to harness the full potential of machine learning in environmental research while avoiding common pitfalls and misinterpretations, ultimately promoting its more transparent and reliable use for scientific discovery and policy support.

## When and Why to Use Machine Learning in Environmental Systems

Environmental modeling has long relied on physics-based approaches that describe the system behavior through governing equations. These models remain preferable when underlying mechanisms are well understood, as they provide interpretable and causally grounded representations. However, traditional models are often limited by unresolved subgrid processes, poorly constrained parameters, and incomplete process representations.^10^ In these contexts, ML provides complementary value by capturing patterns directly from data, improving the representation of unresolved processes, and integrating diverse data sources.^11^ Rather than replacing physics-based models, it is increasingly used alongside them, motivating hybrid approaches that combine physical consistency with data-driven flexibility.^12^

## Integrating Prior Knowledge into Machine Learning

### The Importance of Domain Knowledge in Machine Learning Modeling

Prior knowledge represents the theoretical or mechanistic understanding that exists beyond empirical data.^13^ It aligns model reasoning with causal and physical principles, thereby enhancing interpretability, referring to the extent to which model behavior can be understood and explained, and ensuring that learned relationships remain stable under changing environmental conditions.^14^ Genuine generalization, referring to a model’s ability to perform well under new or unseen conditions, arises not from model complexity, but from embedding such knowledge into the learning process.^15,16^ Such integration goes beyond feature selection, which refers to selecting the input variables used in a model, and instead shapes models to reflect underlying system mechanisms.

This need is particularly acute in environmental systems, characterized by variables that interact through strong couplings, feedback loops, and nonlinear processes across scales.^17^ If physical constraints are not imposed, models may learn patterns that only work under the specific conditions seen in the training data, thereby limiting their generalization capabilities. Incorporating prior knowledge guides learning toward physically plausible behavior rather than purely statistical prediction. In many environmental applications, however, this knowledge rarely exists as explicit equations; instead, it is documented across empirical studies and the domain literature and must be synthesized before it can be incorporated into machine-learning models.

To better integrate domain knowledge into machine learning, physics-informed machine learning (PIML) methodologies have been proposed.^12,18,19^ This approach helps to overcome the limited physical interpretability and poor generalization of purely data-driven models. At the same time, it complements traditional physics-based models, which often depend on simplified parametrizations and require extensive computational resources, thereby limiting their achievable spatiotemporal resolution.^20^ A typical example is a physics-informed neural network (PINN), which enforces known physical laws by incorporating the residuals of partial differential equations (PDEs) into the loss function, which measures the mismatch between model predictions and expected physical behavior, during training.^21^ Conversely, ML can be integrated into general circulation model (GCM) components, replacing or refining traditional parametrizations and enabling data-driven representations of unresolved processes within a physically consistent framework.^22^

However, when governing equations cannot be specified in advance, physical laws may instead be inferred from the data. Sparse system identification methods, such as sparse identification of nonlinear dynamics (SINDy), recover explicit analytical forms of dynamical equations by identifying a parsimonious set of terms from a large library of candidate functions.^23^ As a result, these methods yield interpretable governing equations that are consistent with observed dynamics and remain valid beyond the training regime.^23^ Extensions such as implicit-SINDy further relax structural assumptions by allowing rational and implicit equations, broadening applicability to systems with hidden variables or scale-separated processes.^24^

### The Role of Prior Knowledge Across Environmental Domains

Before introducing domain-specific examples, it is useful to consider how prior knowledge is applied across different environmental fields. The following sections provide representative examples from environmental analysis, toxicology, atmospheric science, and engineering, illustrating how machine learning has been used in each context. Readers can refer to the domain most relevant to their research to gain an overview of the current applications and common practices.

#### Environmental Analysis

In environmental analysis, machine learning serves as a bridge between laboratory measurements and the environmental interpretation. It enables the extraction of meaningful chemical and structural information from complex instrumental data, while simultaneously supporting the reconstruction of large-scale spatial and temporal patterns from limited observations, a process critical for comprehensive environmental exposure assessment.^25,26^

At the analytical scale, ML is increasingly applied across instrumental pollutant analysis, from signal preprocessing and spectral interpretation to compound identification and source or risk assessment. By capturing patterns within complex chromatographic, spectroscopic, and mass spectral data, ML improves analytical efficiency, accuracy, and facilitates the discovery of previously unrecognized contaminants, particularly within nontarget analysis workflows.^27–31^ This practical application demonstrates how ML operationalizes the “extraction of meaningful information” described in the preceding section. However, model performance is often data-dependent. For example, one study found that a model relying only on peak-related features exhibited limited classification ability (PCA explained variance = 46.53%); when test-related parameters such as collision energy (CE) and retention time (RT) were integrated, the explained variance rose to 48.14%, leading to a more distinct separation among samples.^32^ Furthermore, the value of these identifications is amplified by coupling such analytical frameworks with toxicity databases like the EPA CompTox Chemical Dashboard, enabling the linkage of identified chemical structures to their biological effects and thereby strengthening their utility for policy-relevant decision support.^33–35^

At broader environmental scales, the success of spatial prediction in environmental analysis increasingly relies on the ability to compile and harmonize large, representative datasets from heterogeneous sources, such as field monitoring data, literature archives, and open public databases.^36,37^ Recent studies have demonstrated that, while ML can efficiently map pollutants (e.g., fluoride, arsenic, and per- and polyfluoroalkyl substances) at regional to global scales and fill data gaps in areas with limited observations, predictive reliability remains contingent not only on data quality but also on the models’ ability to capture the underlying environmental patterns and mechanisms.^38–42^ However, practitioners should remain aware that, even with increasingly large and diverse datasets, geospatial models remain susceptible to spatial dependence, data imbalance, and unquantified uncertainty.^43^

#### Environmental Toxicology

A deep understanding of biological mechanisms is essential for the meaningful and robust application of ML to environmental toxicology. In nanotoxicology, for instance, basic physicochemical characteristics of nanoparticles (e.g., size and composition) are often insufficient to fully explain their biological effects; therefore, descriptors should be selected rationally according to specific research objectives.^44^ For example, the accurate prediction of protein-corona formation and nanoparticle toxicity depends not only on their surface chemistry but also on biologically relevant contextual conditions, such as the composition of the surrounding biological fluids (e.g., plasma), incubation temperature, and the dispersion environment.^45,46^

Beyond the molecular level, biological responses to environmental pollutants are also shaped by organ-specific distribution patterns and the inherent spatiotemporal heterogeneity of metabolites.^47^ For instance, mass spectrometry imaging has been used to reveal spatial biochemical processes that underlie observed toxic effects.^48–50^ In this context, unsupervised learning techniques offer valuable opportunities for uncovering mechanisms that may not be captured by supervised approaches commonly used in environmental science and engineering.^1,51^

Extending this principle to the environmental scale, ML can predict the spatiotemporal dynamics of contaminant toxicity risks across different media (e.g., water, soil, and air). However, to achieve reliable and predictive models, environmental factors that potentially modulate the toxicity or exposure pathways of pollutants must be explicitly incorporated into the modeling process.^52–54^

#### Atmospheric Science

Among the most common applications of ML in atmospheric sciences is the prediction of fine particulate matter (PM_2.5_) concentrations due to its direct relevance to both air quality management and public health.^55–57^ While models often incorporate standard features like meteorological drivers, emission-related temporal patterns, and land-use characteristics, significant performance gains are achieved by integrating domain-specific predictors, including air mass trajectory information and chemical indicators such as O_*x*_ (NO_2_ + O_3_).^58^ This domain-informed feature selection and construction can substantially enhance model performance, improving the coefficient of determination (*R* ^2^) from roughly 0.62 to above 0.90.^59^ Furthermore, a major strength of ML in this field is its capacity for data fusion. To compensate for data gaps in satellite retrievals and the coarse resolution of outputs from chemical transport models (CTMs), ML approaches integrate outputs from models with satellite, ground-based, and reanalysis data as physical-chemical constraints, thereby significantly improving the accuracy of population exposure assessments.^60–63^

To further improve predictive accuracy, an ensemble learning framework, which combines multiple models to improve predictive performance and robustness, has proven particularly effective.^64^ These frameworks enhance the robustness of atmospheric predictions by reconciling outputs from multiple models and data streams, yielding high-resolution exposure estimates that better capture the spatial and temporal variability relevant to health and policy analyses.^65–67^ Nevertheless, it is imperative to note that model selection should be context-dependent, as ensembles are not always superior to single models.^51^

#### Environmental Engineering

Incorporating industry-specific domain knowledge into model training is highly recommended for water treatment applications.^68^ This integration becomes particularly important when data are limited, as it guides the selection of process-relevant operational and feature variables, thereby enhancing both model robustness and interpretability.^69^ For instance, model performance for THM formation was substantially improved by including specific ultraviolet absorbance (SUVA) as a mechanistically relevant variable, with the model’s *R* ^2^ increasing from 0.92 to 0.97.^70^ In another application, ML was successfully used to optimize a microbubble-enhanced cold plasma reactor, where physically meaningful parameters such as discharge distance and gas flow rate ensured both predictive accuracy and physical interpretability.^71^

In solid waste management, models trained on operational variables derived from mass and energy balances have achieved high accuracy (*R* ^2^ > 0.93) in predicting flue-gas CO_2_ concentrations from municipal solid-waste incineration.^72^ The resulting emission estimates were consistent with both direct measurements and composition-based calculations, demonstrating the effectiveness of process-informed feature selection.^72^ Machine-learning analyses of heterogeneous waste combustion have also shown that elemental composition and moisture content, which are grounded in combustion thermodynamics, are the dominant predictors of combustion behavior.^73^ At the system scale, streamlined life-cycle assessment frameworks integrate conservation laws and defined process boundaries as priors.^74^ These constraints help ensure that ML-based estimates of waste management carbon footprints remain physically consistent and interpretable^74^ (Figure 1).

**Figure 1:**
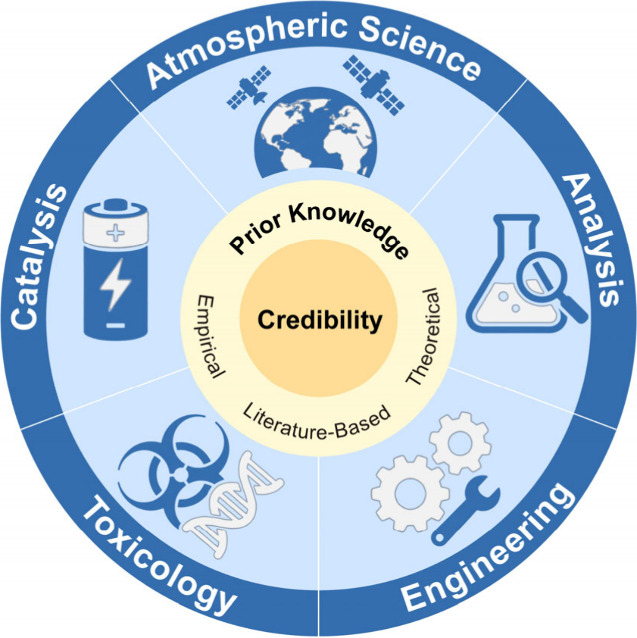
Schematic illustration of how prior knowledge contributes to the credibility of machine learning in environmental research.

## Workflows

### Data Acquisition and Preprocessing

Environmental data exhibit substantial complexity and heterogeneity, typically acquired through multiple pathways. Principal sources encompass multiplatform observations (e.g., in situ monitoring networks and satellite remote sensing), experimental and field measurements (including laboratory analyses and on-site surveys), published literature and curated databases (such as peer-reviewed journals and governmental repositories), and outputs from numerical simulations produced by process-based (non-ML) models.^75^ Given that data obtained from heterogeneous sources frequently exhibit missing values, format inconsistencies, and other irregularities, data preprocessing is required prior to training ML models.

Standard preprocessing procedures include data cleaning, encoding, and normalization.^76^ Data cleaning involves outlier detection and treatment, missing value handling, and the removal of duplicate entries. It is critical to investigate the causes of outliers during their detection and removal, with due consideration given to the potential loss of key information. Unlike traditional modeling approaches, where preprocessing may serve primarily to improve data quality, in ML, these steps can substantially affect model behavior, generalization, and performance evaluation. In particular, preprocessing should be performed without access to validation or test data, as incorporating information from these data into the training process can bias model fitting.

Data encoding transforms categorical variables into numerical representations (e.g., label encoding or one-hot encoding) to enable algorithmic processing.^77^ Normalization and standardization rescale features, particularly for algorithms sensitive to feature magnitude.^78^ A critical consideration in ML workflows is that scaling parameters (e.g., mean and variance) should be derived exclusively from the training dataset and then applied to validation and test data. Applying normalization across the entire dataset prior to data splitting can lead to data leakage, which refers to the unintended use of information from the validation or test set during model training and consequently inflated performance estimates. Careful design of preprocessing pipelines is therefore essential to ensuring valid model evaluation and reproducibility.

Because of differences in their mathematical formulations and inductive mechanisms, ML models exhibit heterogeneous sensitivities to feature scale. Gradient-based learners (e.g., linear regression, logistic regression, and neural networks) and distance-based methods (e.g., *k*-means clustering, *k*-nearest neighbors, and support vector machines) are highly scale-sensitive; without appropriate scaling, high-magnitude features can dominate optimization and distance computations, yielding biased or suboptimal solutions. By contrast, tree-based models (e.g., decision trees, random forests, and gradient-boosted trees) are largely scale-invariant because they partition the feature space via threshold-based splits that are immune to absolute feature magnitudes. Accordingly, in comparative studies spanning multiple model families, the blanket application of normalization or standardization warrants a careful scrutiny.

Dimensionality reduction is commonly applied when the feature space is high dimensional, particularly when multicollinearity, noise accumulation, or limited sample size may impair model stability. Its primary objective is to project data into a lower-dimensional space while preserving relevant structure or information. Different methods rely on distinct assumptions and are suited to different analytical goals.^79,80^ For example, principal component analysis (PCA) seeks orthogonal directions that maximize the variance and is widely used for noise reduction and feature compression. Linear discriminant analysis (LDA) incorporates class labels and aims to maximize class separability, making it suitable for supervised classification tasks. Factor analysis (FA) assumes that observed variables are driven by latent factors and is often used for interpretability. Independent component analysis (ICA) separates statistically independent sources and is useful when the underlying signals are assumed to be non-Gaussian and independent. The choice of method should therefore be guided by the data structure, the availability of labels, and the intended balance between the interpretability and predictive performance.

For studies with sparse observations and limited access to experimental data, the data structure differs from the large-sample settings typical of many machine learning benchmarks. As a result, models are more prone to overfitting, where models capture noise in the training data rather than generalizable patterns and unstable evaluation. In such cases, synthetic or simulation-based data may be used to augment training or support sensitivity analysis.^81^ However, their effectiveness depends critically on how well the simulated data represent the underlying systems. Poorly designed simulations may introduce bias or unrealistic patterns, potentially degrading the model generalizability rather than improving it.

The central rationale of multisource data fusion is to integrate heterogeneous monitoring data streams (e.g., in situ station measurements, remote sensing observations, and model simulations) into a unified, internally consistent high-dimensional dataset via methods such as spatial interpolation, temporal harmonization, and cross-source feature engineering, which refers to constructing or transforming input variables by combining information from multiple data sources. This integration enables the synergistic exploitation of intersource complementarities, thereby enhancing the accuracy and reliability of environmental monitoring. For example, in peatland monitoring, fusing low-cost sensor data, eddy-covariance flux tower observations, chamber-based measurements, and satellite imagery has facilitated the development of more accurate calibration models and improved the reliability of CO_2_ concentration estimates.^82^

### Model Development Process

#### 1 Model Selection

When selecting ML models, priority should be given to the intrinsic requirements of the scientific question and the characteristics of the available data. First, the modeling objective must be precisely defined and mapped to a canonical ML task. For example, land-use and land-cover type identification should be formulated as a classification problem,^83^ whereas pollutant concentration prediction should be framed as a regression task.^84^ Building on this foundation, researchers should conduct an in-depth analysis of the intrinsic spatiotemporal characteristics of environmental data (e.g., spatial autocorrelation in pollutant dispersion processes and temporal dependencies in monitoring records) and, considering practical constraints such as data volume and quality, interpretability requirements, and deployment costs, this analysis can guide the identification of candidate models, which should then be evaluated and compared, as no single algorithm can be assumed to perform best a priori (Figure 2).

**Figure 2:**
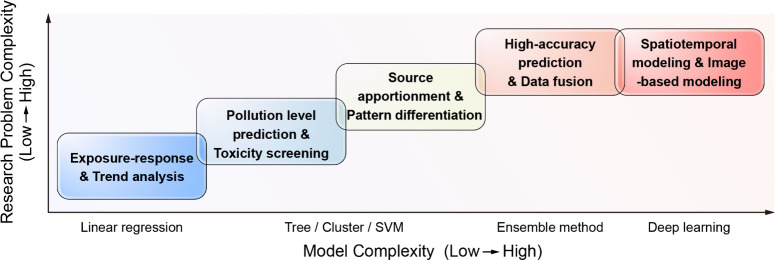
Common environmental research problems across model and scientific problem complexity. The horizontal axis represents increasing model complexity or parametrization, ranging from linear models through nonlinear models to ensemble methods and deep learning approaches. The vertical axis denotes the complexity of the underlying scientific questions.

In machine learning, models differ in interpretability and complexity. To better elucidate a model’s internal mechanisms and predictive behavior, researchers commonly categorize models as black-box, white-box, and gray-box.

Black-box models include ensemble methods such as random forests, gradient boosting trees, XGBoost, as well as advanced algorithms like support vector machines (SVM) and artificial/deep neural networks (ANN/DNN). These models offer strong predictive power arising from complex internal structures and a large number of parameters. However, this does not necessarily imply that more data are always required. Data requirements depend on the complexity of the underlying relationships and the number of model parameters. Overly complex models relative to the available data may lead to overfitting and poor generalization. Accordingly, these approaches are typically applied in settings where the available data are sufficient to support the chosen model complexity. Under such conditions, models can reliably capture complex patterns in the data, and the primary modeling objective often shifts from elucidating precise cause-and-effect pathways to capturing robust correlations between inputs and outputs. For example, XGBoost can be used to identify and predict sedimentary plastic pollution hotspots in urban watersheds, where pollution patterns are jointly driven by topographic, hydrological, and anthropogenic factors.^85^ Here, the model’s high predictive accuracy is prioritized over the interpretability of its specific internal decision processes.

By contrast, white-box models, including regression-based approaches (e.g., linear regression, logistic regression, ridge regression, and LASSO regression), linear discriminant analysis, naive Bayes, and Bayesian additive regression trees (BART), are characterized by transparent structures and clear logic. The interpretability of their parameters and decision paths facilitates the direct analysis of model behavior and validation of results. Because of their transparent computation, these models are typically applied to environmental systems with relatively well-understood mechanisms. For example, Moon and Kim^86^ used correlation-based feature selection with multinomial logistic regression to predict precipitation type; the clearly interpretable coefficients revealed how different meteorological variables influence precipitation phase, providing physically meaningful insights for winter precipitation forecasting. Compared with black-box models, white-box models typically involve fewer parameters, enabling parameter optimization with relatively small datasets while offering higher interpretability. However, the computational nature of these models, which relies on explicit formulation, means that bias can arise in the results whenever the parameters prespecified in the model fail to match the true underlying parameters of the environmental systems.

Gray-box models lie between the two, striving to balance performance and interpretability. They typically integrate interpretable rules or domain-specific physical knowledge with data-driven learning algorithms, thereby improving predictive accuracy while partially exposing the model’s internal computational pathways. Because many domains within environmental systems are data-scarce and their mechanisms are not fully characterized, gray-box modeling has become a significant research focus for such problems. For example, PINNs embed the governing advection-diffusion-reaction system of partial differential equations that describe multispecies contaminant transport directly into the neural network’s loss function. In this way, under data scarcity, PINNs can use physical laws as strong constraints to accurately predict the spatiotemporal distribution of contaminants in groundwater and successfully invert key hydrogeological parameters.^87^

Currently, researchers are integrating black-box and white-box models to improve interpretability, typically following two hybrid frameworks. The first is mechanistic model augmentation, which employs a predominantly mechanistic (white-box) model in which selected hard-to-specify parameters, functional forms, or source terms are replaced or parametrized by a compact ML component. For example, Shen et al.^88^ embedded ML within the Community Atmosphere Model version 6 (CAM6) to develop the MAM4-ML framework, where the ML module dynamically partitions black-carbon (BC)-containing versus BC-free particles, replacing the aerosol module’s simplifying assumption of fully internally mixed black carbon and thereby enabling more accurate simulation of BC aging and optical properties. The second is posthoc bias correction, in which a mechanistic model first generates simulations that are subsequently adjusted by an ML model (e.g., gradient boosting trees or neural networks) trained to capture systematic discrepancies relative to observations. As an illustration, Wu et al.^89^ proposed WeatherGNN, a graph neural network that models complex dependencies among meteorological variables and across spatial regions to learn and correct systematic biases in numerical weather prediction (NWP).

In addition to manual model selection based on domain knowledge, automated machine learning (AutoML) provides a complementary framework for model selection, hyperparameter tuning, which refers to adjusting model settings that are not learned from the data, and ensemble construction.^90^ AutoML systematically evaluates multiple algorithms and parameter configurations to identify high-performing models, supporting rapid benchmarking across model families.^90^ Recent AutoML frameworks also increasingly incorporate interpretability and posthoc explanation tools, allowing their use in applications where transparency is required.^91^

#### 2 Model Construction and Optimization

##### Hyperparameter Tuning

Hyperparameter tuning methods range from unguided to increasingly adaptive strategies. Traditional hyperparameter tuning methods include grid-search and random search. Grid search requires evaluating every combination of hyperparameters, which is straightforward to implement, but becomes computationally prohibitive when the search space is large. Random search, by contrast, explores the hyperparameter combinations via random sampling, which is more efficient than an exhaustive grid search.^92^ However, because their sampling is unguided, both methods can struggle to approach the global optimum within a limited evaluation budget and can be particularly unstable in high-dimensional hyperparameter spaces.

To improve the search efficiency, a range of guided or heuristic optimization methods have been developed, including evolutionary algorithms (e.g., genetic algorithms) and other greedy or adaptive search strategies. These approaches iteratively refine candidate solutions based on prior evaluations and are particularly useful in high-dimensional or irregular search spaces, where simple sampling strategies are inefficient. Bayesian optimization represents a further development. This method uses a probabilistic surrogate model (e.g., a Gaussian process) to approximate the relationship between hyperparameters and model performance and relies on acquisition functions such as expected improvement (EI) to guide the selection of configurations for evaluation. By using information from previous evaluations to guide subsequent searches, this method can identify high-performing hyperparameter regions with far fewer evaluations than random or grid search.^93^

##### Model Training

Prior to model training, the dataset is typically partitioned into training, validation, and test sets. The training set is used for model fitting, the validation set for hyperparameter tuning and model selection, and the test set for final performance evaluation. However, using the training set for both model fitting and hyperparameter selection can lead to overfitting and an overestimation of the model’s true performance.

When data are limited or a more robust performance estimate is required, *k*-fold cross-validation, in which the data are split into multiple subsets and repeatedly used for training and validation, may be employed.^94^ In this approach, the data are divided into *k* subsets that are alternately used for training and validation, providing a more stable estimate of the model performance. However, cross-validation introduces additional computational cost and is not always necessary when sufficient data are available. The choice of *k* reflects a trade-off among dataset size, computational cost, and estimation variance.^95^ Smaller values of *k* are faster but yield higher variance estimates, while larger values are more computationally expensive but provide lower variance estimates.

Another approach to preventing overfitting is early stopping. During iterative training, this mechanism monitors a chosen metric (e.g., loss or accuracy) on a held-out validation set to determine whether training should be terminated prematurely. Specifically, when the validation metric ceases to improve (e.g., when accuracy does not increase, or loss does not decrease) over a prespecified number of consecutive epochs, where an epoch refers to one complete pass through the training dataset, training is halted even if performance on the training set continues to improve. This helps limit the model’s tendency to fit noise and incidental details in the training data, thus improving its generalization capability on unseen data.

##### Model Evaluation

Model evaluation plays a central role in determining whether a model provides reliable and generalizable predictions and should precede interpretability analysis. Within the evaluation framework of ML models, the choice of metrics is not arbitrary; their appropriateness directly determines the validity of the performance assessment and the direction of model optimization. Model quality should be assessed not only by metric values themselves but also by their consistency across datasets and their alignment with the intended application.

Among the most frequently employed metrics, RMSE and *R* ^2^ are the two most commonly used in regression problems, whereas AUC and accuracy are prevalent for classification tasks.^75^ Different metrics characterize model performance from distinct perspectives, and an inappropriate choice can lead to a serious misinterpretation. In classification tasks, precision prioritizes the reduction of false positives, whereas recall emphasizes the minimization of false negatives. In screening potentially toxic substances, high recall is often preferred to avoid missing hazardous candidates, even at the cost of increased false positives.^96^ By contrast, for biomarker identification, high precision is typically favored to reduce false alarms and ensure reliability.^97^ When both error types are of concern, the F1 score provides a balanced evaluation by jointly considering precision and recall.

Beyond metric selection, several common pitfalls should be considered when assessing the model quality. First, different metrics have distinct interpretations and scales. For example, RMSE is expressed in the same units as the target variable, whereas *R* ^2^ is a dimensionless measure of the explained variance. A direct comparison of their magnitudes without considering these differences can therefore be misleading. Second, discrepancies between training and validation or test performances should be examined to identify potential overfitting. Finally, the model performance should not be judged based on a single metric. Instead, multiple criteria should be considered, including predictive accuracy, robustness across data splits, and consistency with the underlying scientific objectives.

##### Interpretability Analysis

While high predictive accuracy is often the primary goal of ML, it does not guarantee that a model has captured meaningful relationships. This issue is particularly critical in policy-oriented research, where model transparency is essential for a credible interpretation and public trust. Consequently, the field of explainable AI (XAI) has emerged to make the logic of complex models more transparent and trustworthy to users. The objective is not to replace powerful models with simpler ones but to augment high-performing models with explainability, thereby enabling reliable, evidence-based decision-making. In practice, interpretability techniques can be approached at two complementary levels: global explanations, which describe how a model behaves on average across an entire dataset, and local explanations, which clarify why the model produces a specific prediction for an individual instance.^98,99^

Partial dependence plots (PDPs) are one of the most widely used tools in global interpretability techniques. By averaging over other predictors, PDPs reveal the overall trend between a continuous feature and the response, such as linear, monotonic, or nonlinear relationships.^100^ However, they describe average model behavior rather than individual predictions and may be biased when strong correlations exist among features.^100^ Similarly, built-in feature importance measures from tree-based models (e.g., random forests, XGBoost) provide another form of global interpretation by quantifying each feature’s overall contribution to model performance. These measures, often calculated from the reduction in a splitting criterion (e.g., Gini impurity) or a permutation technique, quantify each feature’s overall contribution to the model’s performance.

Among local interpretation techniques, local interpretable model-agnostic explanations (LIME) and Shapley additive explanations (SHAP) are two widely used approaches that are increasingly applied to enhance the interpretability of often “gray-box” or previously opaque models.^101–103^ LIME approximates the behavior of a complex model in the neighborhood of a target instance by generating a set of perturbed samples and obtaining their corresponding predictions.^104^ It then fits a simple surrogate model, typically a sparse linear regression, to estimate the relative contribution of each feature within this local region.^104^ In contrast, SHAP provides a game-theoretic formulation that assigns each feature a unique Shapley value, representing its average marginal contribution across all possible feature combinations.^105,106^

By quantifying feature contributions, SHAP helps translate predictive models into mechanistic understanding by clarifying complex interactions across physicochemical, biological, and environmental systems.^107–113^ In parallel, explainable machine learning has been applied to policy-relevant analyses such as determining the major socio-environmental factors driving childhood lead exposure and identifying critical urban characteristics associated with rainstorm risks across Chinese cities.^114,115^ These applications demonstrate how interpretable machine learning not only elucidates underlying environmental mechanisms but also transforms predictive insights into actionable guidance for environmental management and public-health policy.

#### 3 Illustrative Environmental ML Workflows

The overall workflow is illustrated in Figure 3. Two examples of implementing this workflow are considered below.

**Figure 3:**
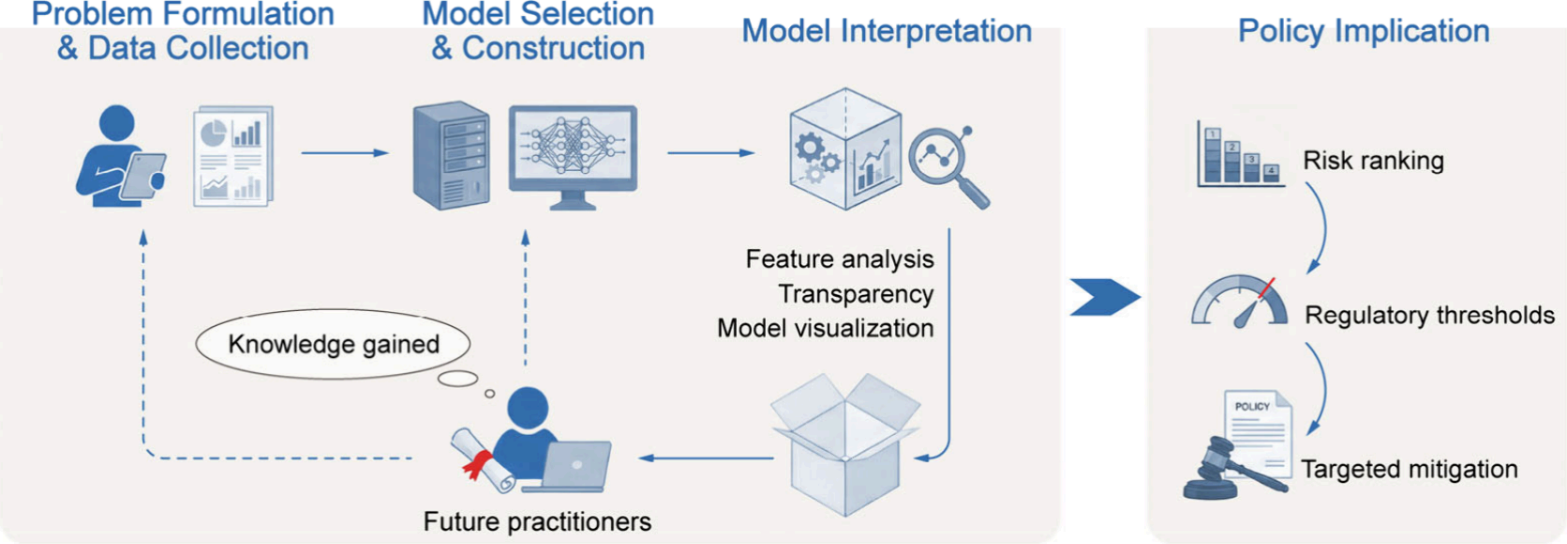
End-to-end workflow for environmental machine learning, from problem formulation to policy implication. The feedback loops highlight how insights from model interpretation guide future data collection, model refinement, and the broader transfer of scientific understanding.

##### Example 1. Ozone Exceedance Prediction

**Data Structure.** Ozone formation is influenced by both meteorological conditions (e.g., temperature, solar radiation, and boundary layer height) and chemical precursors (e.g., NO_2_ and HCHO). These variables are typically continuous, moderately correlated, and subject to environmental variability. In this example, ozone exceedance is defined on the basis of a concentration threshold, and the task is formulated as a classification problem.

**Model Choice Justification.** A logistic regression model is used as an interpretable baseline to assess linear relationships and the directionality of the predictor effects. A random forest model is further introduced to capture potential nonlinear interactions and complex dependencies among variables, allowing a comparison between simple and more flexible approaches.

**Evaluation Strategy.** Given the classification setting and potential class imbalance, model performance is evaluated using ROC-AUC rather than accuracy alone. A stratified train–test split is applied to preserve class proportions and ensure a reliable performance estimation.

**Interpretation.** Interpretation is model dependent. Regression coefficients provide direct insight into feature directionality in logistic regression, whereas feature importance measures in random forests highlight the dominant predictors. Comparing these perspectives helps to assess both consistency and model-specific behavior.

*Reproducibility Note.* A fully reproducible implementation of this workflow, including data generation, preprocessing, model training, and evaluation, is provided in the Supporting Information (Codes S1–S4), enabling readers to explore how workflow design influences model performance and potential pitfalls.

##### Example 2. Toxicity Prediction of Nanoparticles

**Data Structure.** In laboratory-based environmental studies, datasets often consist of physicochemical properties (e.g., particle size, surface chemistry, composition) and experimentally measured biological responses (e.g., cell viability, oxidative stress). These datasets are typically small, heterogeneous, and influenced by both experimental conditions and intrinsic material properties.

**Model Choice Justification.** Given a limited sample size and the need for interpretability, simpler or regularized models (e.g., linear models, decision trees) are often preferred.

**Evaluation Strategy.** Standard train–test splits may be insufficient for small datasets, and cross-validation is commonly applied to obtain stable performance estimates. In addition, alignment between predicted outcomes and experimental end points is critical.

**Interpretation.** Interpretation focuses on linking model outputs to the underlying biological or physicochemical mechanisms. Feature importance, coefficient analysis, or SHAP values can be used to identify key drivers that should be evaluated against existing domain knowledge or experimental evidence.

### Designing Empirical Validation for Model Predictions

While statistical metrics provide an initial assessment of model performance, they do not guarantee that predictions are physically or biologically meaningful in real-world environmental systems. Empirical validation is therefore a critical step to establishing the scientific credibility of machine learning models.

A robust validation strategy should involve independent experimental or observational data that are not used during the model training. One effective approach is to validate predicted intermediate mechanisms before assessing the final outcomes. For example, predicted physicochemical or biological properties (e.g., nanoparticle–protein corona composition) can be experimentally measured using techniques such as liquid chromatography–mass spectrometry and compared quantitatively with model outputs.^45^ Subsequently, downstream biological responses (e.g., cellular uptake, immune activation, or cytokine release) can be evaluated through controlled laboratory assays to confirm whether the predicted mechanisms translate into observable effects.^45^ Validation on systems not included in the training dataset provides a more stringent test of generalizability, particularly when experimental design aligns with the spatial, temporal, and biological scales of the predictions.

### Potential Pitfalls and Biases in Applying Machine Learning to Environmental Research

#### Data

In environmental research, available datasets often overrepresent economically developed regions or dominant demographic groups, while data from underrepresented communities, vulnerable populations, or resource-limited areas remain scarce.^116,117^ Two specific manifestations are particularly common. The first is class imbalance, where samples of critical environmental phenomena (e.g., pollution events and rare species habitats) are extremely underrepresented, posing a significant challenge for models to identify these minority-class instances. The second is spatial heterogeneity, characterized by data points exhibiting clustered distributions across geographic space, resulting in dense coverage in some areas but sparse or absent coverage in others.^43^ To mitigate these issues, sampling strategies should aim to improve representation across classes and regions, and model evaluation should use metrics that are robust to imbalances and spatial bias.

A common but often overlooked issue is data leakage, where information from the test set is inadvertently introduced during the preprocessing or feature construction. This can lead to overly optimistic performance estimates and misleading conclusions. Potential signs of leakage include unexpectedly high model performance, large performance drops when using proper data splitting, or strong predictive power from features that should not be available at the prediction time. Leakage may arise from improper handling of temporal or spatial information such as using future observations or neighboring spatial data during training. As illustrated in Code S5, such leakage can substantially inflate model performance (e.g., *R* ^2^), whereas proper train–test separation that respects temporal or spatial independence (Code S6) leads to a more reliable evaluation. To avoid leakage, all preprocessing steps should be performed using training data only and then consistently applied to validation and test sets.

#### Model

Although ML offers powerful capabilities for analyzing large datasets and capturing nonlinear relationships within complex systems,^45,61,118^ it does not always outperform simpler approaches.^119,120^ In line with the principle of Occam’s razor,^121^ model development should prioritize generalizability and interpretability over unnecessary complexity. In many cases, if a linear or otherwise transparent model achieves comparable performance, there is little justification for adopting a more elaborate machine learning architecture. This principle is illustrated in Code S2, where different models are applied to data generated from a linear relationship. In this setting, a simple model such as logistic regression outperforms a more complex model like random forest (Code S4), highlighting that increased model complexity does not necessarily improve performance when the underlying structure is simple. In practice, this principle should be operationalized by evaluating model performance against appropriate baseline models, which are simple reference models used to assess whether more complex approaches provide meaningful improvements. Such comparisons help determine whether increased complexity leads to meaningful improvements. When simpler models achieve comparable results, they should be preferred, as this can also help mitigate the risks associated with overparameterization.^122^

#### Evaluation

When handling highly imbalanced datasets (e.g., fraud detection or medical diagnosis), the validity of conventional evaluation metrics warrants careful examination.^123^ For instance, although accuracy is widely used, it can be a misleading evaluation metric as a model that always predicts the majority class can still achieve a high accuracy while failing to detect the minority cases of interest. In such scenarios, not only can accuracy be unreliable, but even the commonly used F1-score, which relies on precision and recall can be misleading, whereas metrics such as balanced accuracy and the geometric mean (G-mean) often exhibit greater robustness.^124,125^

This issue is not limited to classification tasks with imbalanced data, but also arises in regression settings. As illustrated in Codes S9 and S10, a time-indexed dataset with a strong smooth trend can yield a high overall *R* ^2^ when modeled using only the time index. However, this apparent performance largely reflects the model’s ability to capture large-scale temporal variation (e.g., seasonal patterns), rather than its ability to predict short-term fluctuations or extreme events that are often of greater practical relevance. When performance is evaluated on subsets of interest (e.g., high-value conditions), model accuracy can degrade substantially. To address this issue, model evaluation should be aligned with the specific objectives of the study. This includes assessing performance on subsets of practical interest (e.g., extreme events), incorporating complementary metrics that capture different aspects of model behavior (e.g., error-based metrics in regression or class-specific metrics in classification), and avoiding over-reliance on a single summary statistic. Such practices provide a more comprehensive and application-relevant assessment of model performance.

#### Interpretation

In the interpretation of model results, feature importance should not be conflated with the model validity or causal influence. When training data are limited or the model is overfitted, seemingly important features may simply capture noise or sampling artifacts.^126^ Even with explainability tools such as SHAP or permutation analysis that quantify the marginal contribution of each variable, these measures remain descriptive rather than causal and thus require considerable caution when extended to policy-relevant interpretations.^127^ Complex models, while capable of reproducing statistical associations with high precision, can often lack mechanistic transparency.^37^ Without grounding in domain knowledge and causal reasoning, such findings risk overstating certainty and leading to interventions that are misguided.

This limitation is particularly evident in settings with limited data or overfitting models. As illustrated in Code S11 and Figure S2, a model trained on a small dataset with additional noise variables can assign non-negligible importance to features that are unrelated to the underlying data-generating process. Despite achieving high performance on the training set, the model exhibits reduced generalization on unseen data, indicating overfitting. Under such conditions, explainability methods such as SHAP faithfully reflect how the model utilizes available features, including spurious patterns, rather than distinguishing the true signal from noise. To mitigate these risks, feature importance analysis should be supported by a robust model validation. This includes comparing importance patterns between training and test sets, assessing stability under resampling or cross-validation, and ensuring consistency with the domain knowledge. Simpler baseline models or regularization, which refer to adding constraints that limit model complexity, can further reduce the influence of spurious features. For policy-relevant applications, importance measures should be complemented with causal analysis or experimental validation to confirm that identified relationships reflect meaningful mechanisms rather than artifacts.

## Conclusions and Perspectives

This Tutorial emphasizes that effective environmental machine learning depends on how data are understood, workflows are rigorously implemented, and predictions are validated in practice. To support this, the Tutorial provides reproducible code examples that readers are encouraged to explore, enabling a clearer understanding of how well-designed workflows can prevent common pitfalls and how improper data handling or workflow choices may lead to them.

Looking forward, advancing environmental machine learning requires not only improving model performance, but also strengthening how models are developed, interpreted, and applied. Recent advances in data-driven tools, particularly large language models (LLMs), are transforming research workflows by accelerating coding and knowledge synthesis. However, their outputs require careful validation due to potential biases, inconsistencies, and outdated information, highlighting the need for rigorous implementation practices. Building on improved workflows, a further challenge is to move beyond purely correlational modeling toward approaches that can better answer scientific and policy-relevant questions.^128–130^ Emerging developments in causal machine learning provide a pathway to quantify intervention effects and address “what if” scenarios that are critical for environmental decision-making.^127,131^ As these capabilities expand, ensuring responsible deployment becomes increasingly important. The integration of high-resolution environmental and human data raises critical concerns regarding fairness, privacy, and transparency.^132–134^ Addressing these challenges will be essential for developing machine learning systems that are not only more effective, but also trustworthy and actionable in real-world environmental applications.^135,136^

## Supplementary Material



## Notes

Additional supplementary code examples (Codes S1–S11) illustrating synthetic dataset generation, machine learning workflows, model training and evaluation, and common pitfalls in machine learning applications; Figures S1 and S2 (PDF)
