# Supplementary material for: A Tutorial on Best Practices and Pitfalls in Applying Machine Learning to Environmental Research
Source: ACS Environ Au. 2026 Jun 5;6(4):553–65. doi: 10.1021/acsenvironau.6c00025 (PMC13377511; doi:10.1021/acsenvironau.6c00025)
Supplement: Supplementary file 1 [file vg6c00025_si_001.pdf]

Supporting Information

for

TUTORIAL

A Tutorial on Best Practices and Pitfalls in Applying Machine Learning to Environmental Research

Zidong Yan[a,b], Jiaqi Li[a,b], Weican Zhang[a,b], Haonan Wen[a], Hao Yu[a], Miao Yu[c], Qian Liu[a,b,d*], Guibin Jiang[a,b]

a State Key Laboratory of Environmental Chemistry and Toxicology, Research Center for Eco-Environmental Sciences, Chinese Academy of Sciences, Beijing 100085, China

b College of Resources and Environment, University of Chinese Academy of Sciences, Beijing, 100049, China

c The Jackson laboratory, 10 Discovery Drive Farmington, CT USA 06032

d Institute of Environment and Health, Jianghan University, Wuhan 430056, China

Email: qianliu@rcees.ac.cn (Q. Liu)

S1. A self-contained worked example

S1.1 Import required packages

Python packages required for data generation, preprocessing, model training, and performance evaluation are first imported. Organizing all required packages at the beginning of the workflow improves transparency and reproducibility. In practice, the exact set of packages will depend on the research question and data type. For beginners, this step is also useful because it clarifies which tools are responsible for each part of the analysis and helps establish a structured workflow before moving on to data processing and modeling. All analyses were conducted in Python (version 3.9.13) using standard scientific computing libraries (NumPy, pandas, scikit-learn, and SHAP). Due to differences in software versions and implementations, minor variations in numerical results (e.g., model performance metrics) may occur across environments, but these do not affect the overall conclusions.

```
import numpy as np
import pandas as pd
from sklearn.model_selection import train_test_split
from sklearn.preprocessing import StandardScaler
from sklearn.linear_model import LogisticRegression
from sklearn.ensemble import RandomForestClassifier
from sklearn.metrics import roc_auc_score
```

Code S1. Import required packages. These packages are used for data manipulation (NumPy, pandas), preprocessing (scikit-learn), model training (logistic regression and random forest), and performance evaluation (ROC-AUC).

S1.2 Generate a synthetic ozone dataset

A synthetic dataset was generated to represent a simplified ozone prediction problem using commonly considered meteorological and chemical variables, including temperature, solar radiation, NO_2, HCHO, relative humidity, wind speed, boundary layer height, and $PM_{2.5}$. These variables were chosen to reflect typical predictors used in atmospheric and environmental modeling.

To ensure interpretability, ozone concentration was constructed using a predefined additive relationship in which higher temperature, stronger solar radiation, and higher precursor concentrations contributed positively to ozone formation, whereas higher relative humidity and stronger wind reduced ozone levels. A random noise term was included to mimic natural variability and measurement uncertainty commonly observed in environmental data.

Finally, a binary outcome variable (high_O_3) was defined based on the upper quantile of the ozone distribution, representing high-ozone events. This transformation converts the problem into a classification task and enables subsequent model comparison under a controlled and transparent setting.

```
np.random.seed(42)
n = 150

temperature = np.clip(np.random.normal(28, 5, n), 10, 40)
solar_radiation = np.clip(np.random.gamma(3, 60, n), 20, 500)
NO2 = np.random.gamma(2.5, 10, n)
HCHO = np.random.gamma(2.0, 4, n)
RH = np.clip(np.random.normal(60, 15, n), 20, 95)
wind_speed = np.clip(np.random.gamma(2.0, 1.2, n), 0.2, 8)
BLH = np.clip(np.random.normal(900, 250, n), 200, 2000)
PM25 = np.clip(np.random.gamma(2.5, 12, n), 5, 180)

O3 = (
    40
    + 1.2 * temperature
    + 0.05 * solar_radiation
    + 0.4 * NO2
    + 0.8 * HCHO
    - 0.5 * RH
```

```
    - 2.0 * wind_speed
    + 0.01 * BLH
    + 0.05 * PM25
    + np.random.normal(0, 8, n)
)

df = pd.DataFrame({
    "temperature": temperature,
    "solar_radiation": solar_radiation,
    "NO2": NO2,
    "HCHO": HCHO,
    "RH": RH,
    "wind_speed": wind_speed,
    "BLH": BLH,
    "PM25": PM25,
    "O3": O3
})

threshold = df["O3"].quantile(0.7)
df["high_O3"] = (df["O3"] > threshold).astype(int)
```

Code S2. Generate a synthetic dataset. This code generates a synthetic dataset with predefined relationships between predictors and ozone concentration and constructs a binary classification target based on a quantile threshold.

S1.3 Explore the dataset

Before model training, it is essential to examine the structure and distribution of the dataset to ensure that subsequent analyses are well grounded.

First, basic inspection of the dataset, including previewing sample rows and checking its dimensions, provides an overview of variable types and sample size. This step helps identify potential issues such as unexpected data formats or inconsistencies.

Second, the distributions of key variables are visualized using histograms. Strong skewness, extreme outliers, or unrealistic value ranges may indicate the need for further data inspection or preprocessing. Depending on the context, this may involve verifying data quality, handling missing values, applying transformations (e.g., log scaling), or selecting models that are less sensitive to distributional irregularities.

Third, the class distribution is examined to assess whether the classification task is balanced. Severe imbalance can lead to misleading performance metrics, as models may favor the majority class. In such cases, alternative evaluation metrics (e.g., precision, recall, F1 score, or ROC-AUC) or strategies such as resampling and class weighting may be required.

Finally, relationships between predictors and the target variable are explored using scatter plots. These visualizations provide an intuitive assessment of potential associations. Weak or unclear patterns may suggest limited predictive value of individual variables, whereas nonlinear trends, clustering, or the influence of extreme observations may indicate the need for feature engineering, nonlinear models, or further data validation.

```
#1 preview the first few rows and dataset size
print(df.head())
print("\nData shape:", df.shape)

import matplotlib.pyplot as plt

#2 visualize distributions of key variables
df[["O3", "temperature", "NO2", "PM25"]].hist(bins=20)
plt.tight_layout()
```

```
plt.show()

#3 check class balance
df["high_O3"].value_counts().plot(kind="bar")
plt.title("Class distribution")
plt.show()

#4 inspect relationship between temperature and ozone
plt.scatter(df["temperature"], df["O3"])
plt.xlabel("Temperature")
plt.ylabel("O3")
plt.show()
```

Code S3. Explore the dataset. This code performs basic exploratory data analysis, including inspecting dataset structure, visualizing variable distributions, assessing class balance, and examining relationships between predictors and the target variable.

S1.4 Train and compare models

The dataset was first divided into training and test sets to enable an unbiased evaluation of model performance. Stratified sampling was applied to preserve the class distribution across splits. Two models were then trained and compared: a logistic regression model as a simple baseline and a random forest model as a more flexible, nonlinear approach. Using a baseline model provides a reference for assessing whether increased model complexity leads to meaningful performance improvements.

Feature standardization was applied to the logistic regression model, as its performance depends on the scale of input variables, whereas the random forest model was trained on the original feature space due to its scale-invariant nature. Importantly, preprocessing steps such as scaling were fitted using only the training data and then applied to the test data to avoid information leakage.

Model performance was evaluated on the test set using the area under the receiver operating characteristic curve (ROC-AUC), which is suitable for binary classification problems and remains informative under moderate class imbalance. In this example, logistic regression achieved slightly higher performance than the random forest model, illustrating that simpler models can be competitive when the underlying relationships are relatively well structured.

```
X = df.drop(columns=["O3", "high_O3"])
y = df["high_O3"]

X_train, X_test, y_train, y_test = train_test_split(
    X, y, test_size=0.25, random_state=42, stratify=y
)

scaler = StandardScaler()
X_train_scaled = scaler.fit_transform(X_train)
X_test_scaled = scaler.transform(X_test)

lr = LogisticRegression(max_iter=1000)
rf = RandomForestClassifier(n_estimators=200, max_depth=4,
random_state=42)

lr.fit(X_train_scaled, y_train)
```

```
rf.fit(X_train, y_train)

print("Logistic regression AUC:", round(roc_auc_score(y_test,
lr.predict_proba(X_test_scaled)[:, 1]), 3)) #0.949
print("Random forest AUC:", round(roc_auc_score(y_test,
rf.predict_proba(X_test)[:, 1]), 3)) #0.919
```

Code S4. Train and evaluate models. This code splits the dataset into training and test sets, applies feature scaling where appropriate, trains two models (logistic regression and random forest), and evaluates their performance using ROC-AUC.

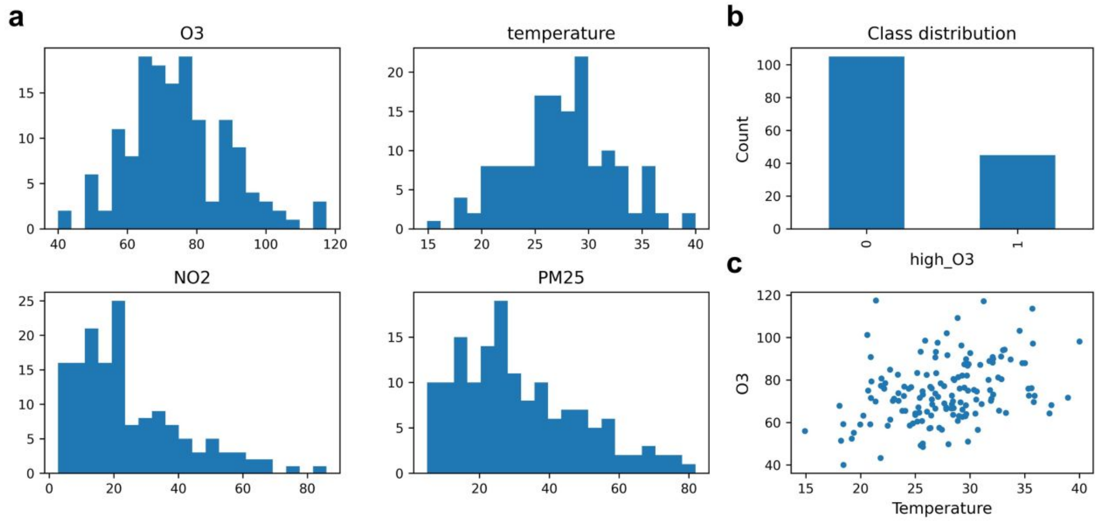

Figure S1. Overview of the synthetic dataset. Distributions of key variables (O_3, temperature, NO_2, and $PM_{2.5}$), class balance for the high-O_3 classification task, and the relationship between temperature and O_3. These plots provide a basic overview of the synthetic dataset.

S2. Common pitfalls in machine learning applications

To clarify the origins of common issues in machine learning applications, synthetic datasets are used to illustrate typical pitfalls in environmental studies, along with corresponding mitigation strategies. The definitions of X and Y depend on the specific research task.

S2.1 Data leakage

Data leakage occurs when information from the test set is inadvertently used during model training, leading to overly optimistic performance estimates and misleading conclusions.

The following example shows a synthetic dataset was generated in which the response variable depends on a continuous predictor and a categorical grouping effect. To mimic common feature engineering practices, a group-level statistic was constructed and included as an additional predictor. However, this statistic was calculated using the full dataset prior to model training, resulting in information leakage.

Such situations are common in environmental applications, for example when site-level averages, spatial aggregates, or future observations are used in feature construction without respecting data partitioning. This can substantially inflate model performance by introducing information that would not be available at prediction time.

```
import numpy as np
import pandas as pd
from sklearn.model_selection import train_test_split
from sklearn.linear_model import LinearRegression
from sklearn.metrics import r2_score

np.random.seed(0)

# generate synthetic data
n = 100
group = np.random.choice([0,1,2,3], n)
x = np.random.randn(n)
y = 5*x + group*2 + np.random.randn(n)

df = pd.DataFrame({"x": x, "group": group, "y": y})

# data leakage: group mean computed using full dataset
```

```
df["group_mean_y"] = df.groupby("group")["y"].transform("mean")

X = df[["x", "group_mean_y"]]
y = df["y"]

X_train, X_test, y_train, y_test = train_test_split(X, y,
random_state=0)

model = LinearRegression().fit(X_train, y_train)
pred = model.predict(X_test)

print("With leakage R²:", round(r2_score(y_test, pred), 3))
```

Code S5. Data leakage through improper feature construction. This code demonstrates data leakage by computing group-level statistics using the full dataset before splitting into training and test sets.

To address this issue, the dataset was first divided into training and test subsets. All group-level statistics were then computed using the training data only and subsequently applied to both subsets. This resulted in a decrease in performance (R^2 decreased from 0.95 to 0.90), indicating that the initial results were affected by data leakage.

```
train_df, test_df = train_test_split(df, random_state=0)

mean_map = train_df.groupby("group")["y"].mean()

train_df["group_mean_y"] = train_df["group"].map(mean_map)
test_df["group_mean_y"] = test_df["group"].map(mean_map)

X_train = train_df[["x", "group_mean_y"]]
y_train = train_df["y"]
X_test = test_df[["x", "group_mean_y"]]
y_test = test_df["y"]

model = LinearRegression().fit(X_train, y_train)
pred = model.predict(X_test)

print("No leakage R²:", round(r2_score(y_test, pred), 3))
```

Code S6. Prevent data leakage using training-only feature construction. This code avoids data leakage by computing group-level statistics using only the training data and applying them consistently to both training and test sets.

S2.2 Spatial autocorrelation

Spatial autocorrelation arises when observations that are geographically close exhibit similar values, violating the assumption of independent and identically distributed samples. When not properly accounted for, this can lead to overly optimistic estimates of model performance.

For illustration, we generated a synthetic spatial dataset by assigning random coordinates to each sample and defining the response variable as a smooth function of location with added noise. This construction induces strong similarity among nearby observations. A standard random train-test split was then applied.

Under random splitting, spatially proximate samples are likely to appear in both the training and test sets. As a result, the model can effectively learn from neighboring observations that are highly similar to those in the test set, leading to inflated performance estimates. This situation commonly occurs in environmental applications, for example when nearby monitoring stations share similar pollution levels or meteorological conditions.

```
import numpy as np
from sklearn.model_selection import train_test_split
from sklearn.ensemble import RandomForestRegressor
from sklearn.metrics import r2_score

np.random.seed(0)

# generate spatial data
n = 200
x = np.random.uniform(0, 100, n)
y = np.random.uniform(0, 100, n)

# smooth spatial field
z = 0.05*x + 0.03*y + np.sin(x/20) + np.random.randn(n)*0.2

X = np.column_stack([x, y])

# random split
X_train, X_test, y_train, y_test = train_test_split(X, z,
random_state=0)

model = RandomForestRegressor().fit(X_train, y_train)
```

```
pred = model.predict(X_test)

print("Random split R²:", round(r2_score(y_test, pred), 3))
```

Code S7. Spatial autocorrelation with random train-test split. This code demonstrates how a random train-test split can lead to overly optimistic performance when spatial autocorrelation is present.

To obtain a more realistic evaluation, the data were split based on spatial regions, ensuring that geographically distinct areas were assigned exclusively to either the training or test set. Under this setting, model performance decreased compared to the random split (R^2 decreased from 0.95 to 0.79), suggesting that random splitting may overestimate performance when spatial autocorrelation is present.

```
train_mask = x < 50
test_mask = x >= 50

X_train, X_test = X[train_mask], X[test_mask]
y_train, y_test = z[train_mask], z[test_mask]

model = RandomForestRegressor().fit(X_train, y_train)
pred = model.predict(X_test)

print("Spatial split R²:", round(r2_score(y_test, pred), 3))
```

Code S8. Spatially structured train-test split. This code applies a spatial split by separating samples into distinct geographic regions for training and testing.

S2.3 Limitations of R^2 for environmental prediction tasks

The coefficient of determination (R^2) is widely used to evaluate model performance in regression tasks. However, in environmental prediction problems, it may not fully reflect model performance under conditions of practical relevance.

To illustrate this limitation, a synthetic time-indexed dataset was constructed in which the response variable is dominated by a smooth large-scale trend, with smaller fluctuations superimposed. A simple model using only the time index as a predictor was able to capture most of the large-scale variation and therefore achieved a relatively high overall R^2.

However, this high performance primarily reflects the model's ability to reproduce broad temporal patterns (e.g., seasonal variability), rather than its ability to capture short-term variability or extreme events (e.g., pollution episodes), which are often of greater importance for environmental risk assessment and decision-making.

```
import numpy as np
from sklearn.linear_model import LinearRegression
from sklearn.metrics import r2_score, mean_absolute_error

np.random.seed(0)

# generate time series with strong trend
n = 200
t = np.arange(n)

trend = 0.1 * t + 5 * np.sin(t/20)
noise = np.random.randn(n)
y = trend + noise

# model using time only
model = LinearRegression().fit(t.reshape(-1,1), y)
pred = model.predict(t.reshape(-1,1))

print("Overall R²:", round(r2_score(y, pred), 3))
```

Code S9. High R^2 driven by large-scale trends. This code demonstrates how a simple model can achieve a high overall R^2 by capturing large-scale temporal trends, despite limited ability to represent short-term variability.

To improve the evaluation, model performance was further assessed on subsets of the data corresponding to higher response values and using error-based metrics that are more sensitive to prediction accuracy. This provides a more informative assessment of model behavior and avoids over-reliance on a single aggregate metric.

```
mask = y > np.percentile(y, 70)

print("High-value R²:", round(r2_score(y[mask], pred[mask]), 3))
print("High-value MAE:", round(mean_absolute_error(y[mask],
pred[mask]), 3))
```

Code S10. Subset-based and error-based evaluation. This code evaluates model performance on a high-value subset of the data and complements R^2 with an error-based metric (MAE).

S2.4 SHAP Limitations of feature importance under overfitting (SHAP example)

Feature importance methods such as SHAP are widely used to interpret machine-learning models. However, these methods explain how a model makes predictions, rather than whether the model has learned meaningful relationships.

To illustrate this issue, a synthetic dataset was constructed based on the ozone prediction example in Section S1, with additional noise variables included as predictors. A flexible random forest model with minimal regularization was then trained, increasing the risk of overfitting.

Under these conditions, the model may capture spurious patterns present in the training data, including those associated with noise variables. As a result, feature importance methods such as SHAP may assign non-negligible importance to variables that are not causally related to the response, as shown in Figure S2.

This issue is particularly relevant in environmental applications, where datasets are often limited in size and may contain correlated or noisy predictors.

```
import numpy as np
import pandas as pd
import matplotlib.pyplot as plt
import shap

from sklearn.model_selection import train_test_split
from sklearn.ensemble import RandomForestClassifier
from sklearn.metrics import roc_auc_score

np.random.seed(42)
n = 120

temperature = np.clip(np.random.normal(28, 5, n), 10, 40)
NO2 = np.random.gamma(2.5, 10, n)
RH = np.clip(np.random.normal(60, 15, n), 20, 95)
wind_speed = np.clip(np.random.gamma(2.0, 1.2, n), 0.2, 8)
solar_radiation = np.clip(np.random.gamma(3, 60, n), 20, 500)

O3 = (
    1.2 * temperature
    + 0.5 * NO2
```

```python
    - 0.6 * RH
    + 0.1 * solar_radiation
    - 0.8 * wind_speed
    + np.random.normal(0, 10, n)
)

threshold = np.percentile(O3, 70)
high_O3 = (O3 > threshold).astype(int)

df = pd.DataFrame({
    "temperature": temperature,
    "NO2": NO2,
    "RH": RH,
    "wind_speed": wind_speed,
    "solar_radiation": solar_radiation,
    "noise_1": np.random.randn(n),
    "noise_2": np.random.randn(n),
    "noise_3": np.random.randn(n),
    "noise_4": np.random.randn(n),
    "noise_5": np.random.randn(n),
    "noise_6": np.random.randn(n),
    "noise_7": np.random.randn(n),
    "noise_8": np.random.randn(n),
    "high_O3": high_O3
})

X = df.drop(columns=["high_O3"])
y = df["high_O3"]

X_train, X_test, y_train, y_test = train_test_split(
    X, y, test_size=0.3, random_state=42, stratify=y
)

rf = RandomForestClassifier(
    n_estimators=400,
    max_depth=None, # overfitting
    min_samples_split=2,
    min_samples_leaf=1,
    random_state=42
)

rf.fit(X_train, y_train)

train_auc = roc_auc_score(y_train, rf.predict_proba(X_train)[:, 1])
```

```python
test_auc = roc_auc_score(y_test, rf.predict_proba(X_test)[:, 1])

print("Train AUC:", round(train_auc, 3))
print("Test AUC:", round(test_auc, 3))

explainer = shap.Explainer(rf, X_train)
shap_values = explainer(X_test)

shap.plots.beeswarm(shap_values[:, :, 1])
shap.plots.bar(shap_values[:, :, 1])

shap_importance = pd.DataFrame({
    "feature": X.columns,
    "mean_abs_shap": np.abs(shap_values.values[:, :, 1]).mean(axis=0)
}).sort_values("mean_abs_shap", ascending=False)

print(shap_importance)
```

Code S11. SHAP importance under overfitting with spurious features. This code demonstrates how a flexible model trained on a dataset with noise variables can assign non-negligible SHAP importance to spurious features.

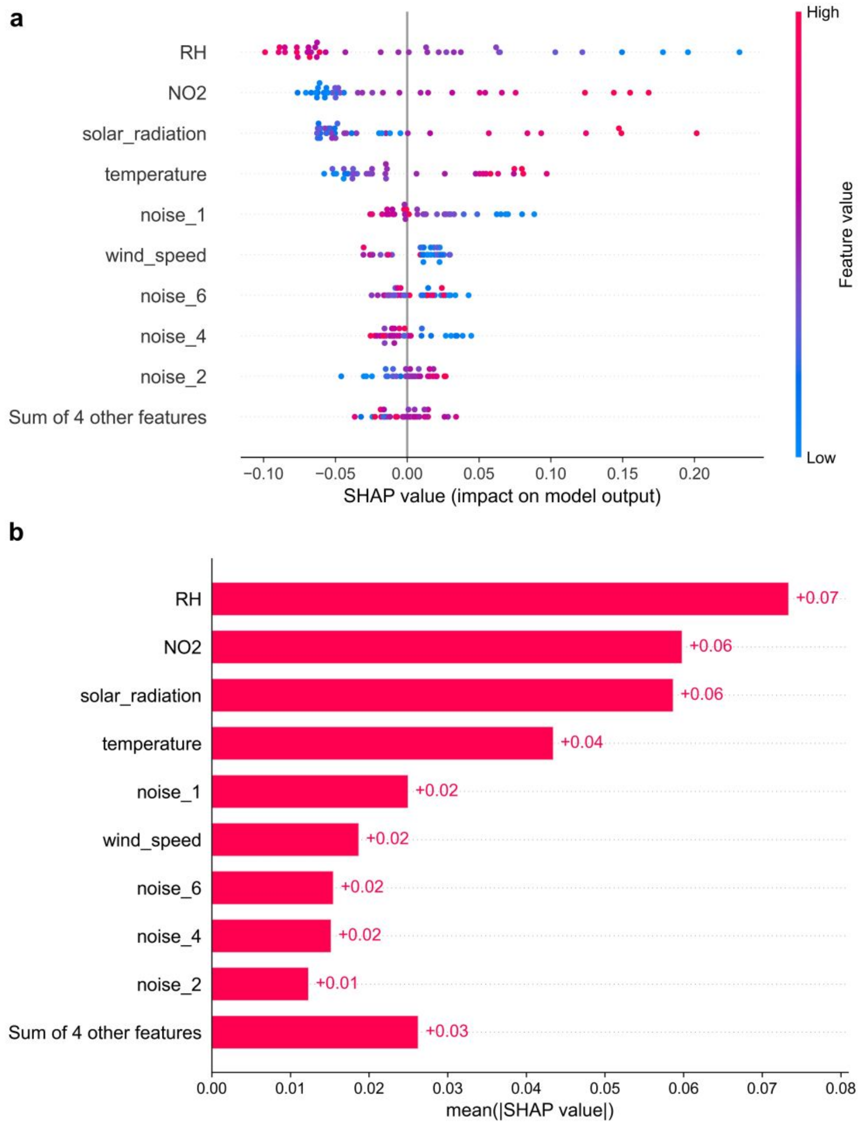

Figure S2. SHAP importance with noise variables. SHAP summary (a) and mean absolute SHAP values (b) for a classification model trained on a small dataset with additional noise variables. Despite having no relationship with the data-generating process, several noise features (e.g., noise_1, noise_6) show non-negligible importance. The model achieves near-perfect performance on the training set but lower performance on the test set, indicating overfitting. Under this condition, SHAP reflects how the model uses features, including spurious patterns, rather than distinguishing true signal from noise.
